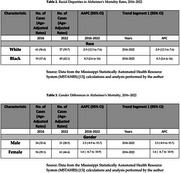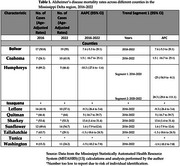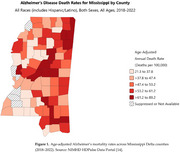# Trends in Alzheimer's Disease Mortality in the Mississippi Delta, 2016‐2022

**DOI:** 10.1002/alz70861_108632

**Published:** 2025-12-23

**Authors:** Nafiseh Gavari

**Affiliations:** ^1^ Jackson State University, Jackson, MS USA

## Abstract

**Background:**

Alzheimer’s disease (AD) is a leading cause of death among older adults in the United States, disproportionately affecting vulnerable populations. In Mississippi—particularly in the rural, predominantly Black Mississippi Delta—AD mortality rates are among the highest in the nation. However, limited research has examined temporal trends and demographic disparities in this region. This study aimed to assess trends in AD mortality rates in the Mississippi Delta from 2016 to 2022, disaggregated by race, gender, and county, to inform equitable public health interventions.

**Method:**

This trends study used age‐adjusted mortality data for individuals aged 65 and older from the Mississippi Statistically Automated Health Resource System (MSTAHRS). Joinpoint regression was employed to estimate Annual Percent Change (APC) and Average Annual Percent Change (AAPC) in AD mortality rates, with 95% confidence intervals. Analyses were stratified by race (Black, White), gender, and Delta County. Publicly available, de‐identified data were used; thus, institutional review board approval was not required.

**Result:**

From 2016 to 2022, AD mortality rates declined among White individuals (AAPC = ‐2.9), while significantly increasing among Black individuals (AAPC = 8.3; 95% CI: 2.6–16.0). Gender‐specific trends showed slight, non‐significant increases for both males and females. County‐level analysis revealed stark disparities, with some counties (e.g., Sharkey and Quitman) showing increases of over 10% in mortality rates, while others (e.g., Humphreys and Leflore) exhibited declining trends. Racial disparities in mortality were more pronounced than gender disparities.

**Conclusion:**

This study highlights critical racial and geographic disparities in AD mortality in the Mississippi Delta. The significant increase in mortality among Black seniors suggests structural inequities in healthcare access, diagnosis, and chronic disease management. Findings support the urgent need for culturally tailored outreach, expanded dementia care infrastructure, and public health policy reforms to reduce AD‐related mortality in underserved rural communities.